# Infringement of the right to surgical informed consent: negligent disclosure and its impact on patient trust in surgeons at public general hospitals – the voice of the patient

**DOI:** 10.1186/s12910-019-0407-5

**Published:** 2019-10-28

**Authors:** Gillie Gabay, Yaarit Bokek-Cohen

**Affiliations:** 1 Behavioral Sciences and Psychology, College of Management Academci studies, 7 Rabin Blvd, 97150 Rishon Letzion, Israel; 2 Nursing Sciences, Tal-Aviv Jaffa Academic College, 7 Rabin Blvd, 97150 Rishon Letzion, Israel

**Keywords:** Communication, Disclosure, Informed consent, Infringement, Negligence, Patient-surgeon trust, Respect for autonomy, Subjective standard

## Abstract

**Background:**

There is little dispute that the ideal moral standard for surgical informed consent calls for surgeons to carry out a disclosure dialogue with patients before they sign the informed consent form. This narrative study is the first to link patient experiences regarding the disclosure dialogue with patient-surgeon trust, central to effective recuperation and higher adherence.

**Methods:**

Informants were 12 Israelis (6 men and 6 women), aged 29–81, who underwent life-saving surgeries. A snowball sampling was used to locate participants in their initial recovery process upon discharge.

**Results:**

Our empirical evidence indicates an infringement of patients’ right to receive an adequate disclosure dialogue that respects their autonomy. More than half of the participants signed the informed consent form with no disclosure dialogue, and thus felt anxious, deceived and lost their trust in surgeons. Surgeons nullified the meaning of informed consent rather than promoted participants’ moral agency and dignity.

**Discussion:**

Similarity among jarring experiences of participants led us to contend that the conduct of nullifying surgical informed consent does not stem solely from constraints of time and resources, but may reflect an underlying paradox preserving this conduct and leading to objectification of patients and persisting in paternalism. We propose a multi-phase data-driven model for informed consent that attends to patients needs and facilitates patient trust in surgeons.

**Conclusions:**

Patient experiences attest to the infringement of a patient’s right to respect for autonomy. In order to meet the prima facie right of respect for autonomy, moral agency and dignity, physicians ought to respect patient’s needs. It is now time to renew efforts to avoid negligent disclosure and implement a patient-centered model of informed consent.

## Background

Surgeons have a duty to conduct a surgical informed-consent discussion with patients prior to surgery. The autonomy to authorize (or not) a certain procedure implies that the term ‘informed consent’ can be aptly termed ‘informed choice’ and is the bedrock principle of medical ethics [1–4]. Surgeons adhere to three criteria of adequate informed consent (IC henceforth); namely: Capacity, autonomy, and disclosure. ‘Capacity’ relates to the patient’s mental ability to make decisions. ‘Autonomy’ is demonstrated by obtaining IC [5]. ‘Disclosure’ relates to the information required to enable the patient to make a decision regarding the procedure [5]. Historically, the idea of IC has been understood in terms of freedom (from interference and paternalism) and patient autonomy [6].

A meaningful IC process constitutes: Patients’ competence to understand, choose, and freely decide; proper disclosure of relevant information, recommendations, and understanding; and eventually, the consent elements are the decision made by the patients in favor of a plan for medical treatment and their authorization to pursue it [1]. Although IC is an integral part of clinical practice, its current doctrine remains mostly a matter of law and ethics. Previous studies and meta-analyses indicate that, in different countries, the content of surgical IC failed to meet acceptable standards, thereby reducing the quality of the surgical IC process and resulting in patients’ inadequate understanding, and consequently, their inability to participate in decision-making [7–12].

Empirical research based on patient perception of IC is scant [13]. This empirical study focuses on patients’ experiences with IC. It explores experiences of participants who signed surgical IC at public Israeli hospitals before undergoing life-saving surgeries due to heart disease, neurological disorders, cancer, and life-threatening accidents. The Israeli Patients’ Rights Law of 1996 (clause 13) [14] a well as the North American and the South African National Health Act (2003; Ch. 2, section 6) [15], hold surgeons responsible for acting upon principles of medical ethics that emphasize the importance of complete and accurate information that patients should receive before surgery to enable their informed decision-making regarding that surgery. Disclosure requires sufficient, relevant, updated information about the diagnosis; the prognosis; the procedure; its aims; its benefits; its ramifications; risks and success rates; and side effects, including pain, discomfort, alternative treatments. The limits of this relevant knowledge are also to be conveyed to the patient [2].

The current law has refined the practice of IC. If patients sign the IC form without a discussion or conversation with the physician, it invalidates the process and is considered by law to be a case of negligence. Furthermore, even cases where patients received inappropriate disclosure are viewed by the law as negligence [16]. The Israeli law [14] requires that only the surgeon conduct the discussion with the patient and document it in writing. Finally, by law, the surgeon is to provide the information voluntarily rather than wait for the patient’s questions.

An effective IC process requires the surgeon to understand patient values and preferences by discussing which risks and benefits of the procedure in question could be especially relevant for each patient [2]. In order to evaluate risks and benefits of treatments the disclosure process should combine inputs of surgeons with those of patients [17]. Surgeons’ training, knowledge, expertise, professional values, that are shaped by informed public attitude, lead to an understanding of available treatments and should be combined with patients’ knowledge of their subjective aims, values and circumstances [17].

Thus, an adequate process of IC entails the need to get to know the patients, their moral beliefs and social structures [6]. Only then is the surgeon able to support the patient in weighing possible benefits and harms and in making a decision of substantial autonomy [6]. McKneally, Ignagni, Martin and D’Cruz [18] claim that attending unresolved patient residual doubts and fears, facilitate patient trust in the surgeon, as IC discussions can help set aside patients’ fears regarding complications and outcomes. The patient may feel that the surgeon understands and respects her concerns and is actively working accordingly [19]. Despite the legal guidelines with regard to conducting IC discussions of patients and physicians, the research literature originating from different Western health systems, shows that these dialogues are deficient, hence, patients’ needs often remain unmet [20–22].

Furthermore, patients may also waive their right to disclosure. In an empirical study of the meaning of the IC form, it was found that patients rated the readability of IC documents and their understanding of them as low. The study evaluated the readability of IRBs IC forms by assigning a score on the basis of the minimal level required to read and understand English text, and found that readability standards are not met and are not suited to the reading comprehension skills of large parts of the population [23]. The extent and timing of disclosure and of signing the IC form may also inhibit or encourage patient-surgeon trust.

Patient trust is essential in determining the level of recuperation, the level of medication adherence and number of readmissions [24–33]. Trust, however, has not been applied to the context of surgical IC. The concept of patient trust includes inter alia, compatibility between the patient’s prior expectations and the behaviors acted upon, a risk evaluation, and the willingness to become dependent on another person [34]. This conceptualization of Hupcey, Penrod, and Morse [35] suggests that the patient may have her own expectations for receiving care and she agrees to be involved in a relationship that may elevate her vulnerability owing to her reliance upon the physician [36, 37].

Extensive studies have examined communication that promotes patient trust in physicians. It was found that trust was higher when technical competence and listening skills, as well as honesty and confidentiality, were characteristics displayed by the physician [38–41]. Physicians’ communication styles were a pivotal prerequisite for the patient to become more involved in the recovery process [42–44]. When patients were involved in decision making to a lesser degree than they would have liked, the effect on trust was more detrimental than if the opposite situation occurred and they felt they had been involved too much [45, 46]. Both physicians and patients perceived the amount of explanations provided by the physician as a measure of the quality of the physician-patient communication [47]. Patient satisfaction with clinical outcomes improved patient trust in the physician [48]. Perceptions and interpretations of patients regarding their encounters with their physicians affected patient satisfaction [47]. Patient empowerment through physicians’ explanations in acute-care units built patient trust and resulted in improved outcomes and in greater well-being, post-discharge [49].

Patient-physician interactions that created doubt, irritation, anxiety, fear, or similar negative feelings developed into distrust [50]. Distrust in physicians was associated with a patient’s feeling of objectification, failure to preserve a patient’s self-value, a physician’s lack of a bedside manner, and lack of cultural competency [49, 51]. Patient distrust has been found to be correlated with more incidences of psychopathology and with lower life satisfaction [52].

Meeting patient expectations was a pivotal theme that combined all categories of trust [35]. Patients evaluated their encounters with their physicians in reference to their prior expectations [34]. Unmet expectations have been found to be correlated with low satisfaction [53–55] and may, therefore, diminish trust. While physicians perceived their explanations as sufficient to meet patient expectations, thinking there were no more important issues to discuss, patients often thought differently about the explanations and were accordingly unsatisfied with the level of explanations [56]. If patient’s expectations are met or even exceeded, then trust in physician is established. However, if the patient is disappointed, because her expectations are not met, distrust is formed. Despite the fact that surgical IC dialogues are intended to promote the patient’s best interest and have much potency in creating surgeon-patient trust, they lack the component of an interpersonal interaction between the surgeon and the patient [6].

This study is the first to link experiences of patients with surgical IC and patient trust in surgeons. As they described their hospitalization experience, participants talked relatively extensively about the process of surgical IC, which is the focus of this study. The research question is: Does patient experience of surgical IC accord with principles of IC that surgeons are instructed to implement? A related question is: How does the conduct of the surgical IC process shape patient trust?

## Methods

We embarked on a qualitative study, which is the first narrative study, to our knowledge, to focus on patients’ experiences of IC. We believe the method is preferable over standard quantitative questionnaires, because qualitative studies are particularly well-suited for understanding patient values, perspectives, experiences, and contextual circumstances – all of which are concerns of medical ethics [57].

### Participants and recruitment

Following ethics approval by the ethics committee of the COLLMAN’S research authority (#099), a maximum-variation approach was employed to recruit participants, enabling the inclusion of a wide range of perspectives. Informants were 12 Israelis who underwent life-saving surgeries in three large public hospitals (1200–3200 beds) and two medium hospitals (700–1200 beds) across the country, with diversity in participants’ age (29–81), gender (6 men, 6 women) and socio-demographic traits. The sample size was determined by the principle of “information saturation”, as participants described the informed consent process and deficient communication the same way, despite the variances in the type of surgery or the hospital size and location [58–61]. Despite the size of the sample, participants fulfill Lincoln and Guba’s (1985) requirement [62] for a wide arrange of attributes (gender, age, disease, hospital). Ten participants were married, one was single, and one was divorced. Table 1 presents demographic and health attributes of participants.

**Table 1:** Participants’ socio-demographic data and the type of disease

Pseudonym	Age Range	Disease	Hospital Size	Profession & status
Michael	30s	cancer Spinal	Large	Software engineer; employed
Gershon	30s	Neurological	Large	Engineer; self-employed
Danit	30s	Limb injuries	Medium	Dancer; self-employed
Ella	30s	Uterine Cancer	Large	Designer; self-employed
Anat	40s	Breast cancer		Teacher; employed
Ron	50s	Limb injuries	Medium	Architect; self-employed
Alona	60s	Sternum cancer	Large	Consultant; self-employed
Itamar	50s	Lung cancer	Medium	Photographer; employed
Joseph	70s	Neurological	Large	Retired
Martin	70s	Neurological	Large	Insurance agent; employed
Michelle	70s	Uterus Cancer	Large	Psychotherapist; self- employed
Yonatan	80s	Heart	Large	Retired

A snowball sampling was used to find Informants in their initial recovery phase upon discharge from a public general hospital. Participants provided contact information about prospective participants from among their acquaintances (i.e., either people they met at the hospital or people whom they heard had just been discharged from a lengthy hospitalization). This method was used since it was difficult to recruit participants upon their discharge from a lengthy hospitalization in acute care and to interview them when they were in a relatively poor physical and emotional state. We focused on participants who underwent major surgeries to emphasize the fact that although their clinical outcomes improved and their lives were saved, they vividly remembered the informed-consent process and were deeply troubled by it. In all interviews regarding their hospitalization experience, the deficient informed-consent process took up much space in their narratives and left a serious imprint on their recollections.

Two narrative interviews were conducted with each participant. Narrative interviews ranged in length from 90 min to 2 hours due to physical discomfort or emotional stress that required breaks. Participants were assured confidentiality and that their participation would have no influence on their future treatments at the hospitals. Participants were asked to sign a written statement of IC regarding participation and publication. They were informed that they could stop the interview at any point they choose. Interviews were conducted in Hebrew, audio-taped, transcribed verbatim, including all emotional expressions, non-verbal utterances and pauses and translated into English. All identifying details, except for participant’s gender and age at the time of interviews, were omitted from the findings section to ensure anonymity. Participants’ names were replaced with pseudonyms to avoid identification.

### Research quality assurance

In order to advance the credibility of this study and allow participants to talk freely and refrain from downplaying or over-emphasizing their feelings, the first author endeavored to create a relaxed and empathetic atmosphere during the interview. In order to enhance the transferability of the conclusions, the first author described the research methodology in detail and provided detailed descriptions of participants’ perspectives. Moreover, the preliminary results were triangulated by a qualitative peer debriefing with eight clinicians and three colleagues who specialize in qualitative methodology. Based on their comments, technical medical terms were clarified and data analysis was enhanced*.*

### Procedures

Twenty-four interviews were conducted at the interviewees’ homes. The issue of surgical IC appeared to be very emotionally loaded. The first interview with each participant was carried out within the first 2 days after discharge and the second interview was carried out a month later. In accordance with the method of a narrative interview, participants were asked to answer one question in detail: “Please tell me how you arrived at the hospital and what you experienced there.” Interviewees spoke of their experiences from the first appearance of symptoms until the time of discharge from hospital. The first author introduced herself as a person specializing in health psychology, studying the experience of hospitalization and explained the research methodology. Almost all interviewees stressed that, although it was very challenging for them to submit to the interview in their poor physical state so soon after discharge, they nevertheless wanted to share their experience in order to improve the experiences of others. The first author thanked them for their willingness to contribute to the project.

The first author actively listened beginning with the opening question, as guided by the narrative method [63], and did not attempt to ask questions, comment, or judge what interviewees said, in order to allow them to express themselves freely and convey their own subjective interpretations for what they remembered had occurred. All were highly emotional.

### Analytic strategy

Data were interpreted using the narrative method, which is based on the assumption that narratives constitute a main cognitive scheme in human development [63–65]. Cases where there were repeated elements across narratives reflect the authenticity of these narratives. The data analysis was informed by the holistic principle that views narratives as representing whole experiences where parts of a narrative are related to each other. For example, openings of narratives that may be related to their endings with pivots of content are expected to be interrelated; and episodes that may seem unrelated at first sight may later be revealed to be associated to one another [66, 67]. The principle of wholeness builds on constructive ontology and epistemology [65].

The data analysis method is based on four analytical phases and is hence suitable for a data-driven analysis and for exploring themes that originate in experiences of participants [68]. In the first phase, the transcription of each interview was read again and again as a whole unit. Initial themes were detected for each participant’s experience. Original words, body language and tone, were documented. In the second phase, each transcribed interview was analyzed using six selection mechanisms as described below [65]; these mechanisms are assumed to describe what participants inadvertently chose to tell and not to tell, leading to the ‘end-point’ of each narrative (i.e., the focal theme).

The selection mechanisms that were at work in the interpretation of the narratives: inclusion - meaning the facts and experiences reported by each participant and the common motif amongst them; sharpening - meaning the events which the participants themselves stated as being central; omission – meaning the occurrences that participants thought were irrelevant to the desired end-points; silencing - meaning those occurrences that participants felt to be in conflict with the desired end-point; flattening - meaning the reductionist stance towards occurrences that participants perceived as unimportant for them; and, attribution of appropriate meaning - relating to meaning ascribed to occurrences that participants found to be compatible with the end-points, although these end-points may not necessarily align with their original meaning.

In the third step, the end-point of each interview was identified, as it emerged from the analysis, using selection mechanisms. In the fourth and last step, the analysis shifted from the individual level to the group level. Themes in the transcribed interviews were read, with attention paid to elements in the process of surgical IC, deficiencies in IC and patient trust.

## Results

The findings, based on patient experiences with surgical IC, indicate deficiencies in the information disclosure, recommendations, and understanding before participants underwent life-saving surgery. All participants related to the communication with the surgeon about the surgery, or lack thereof in their narratives on the hospitalization experience. Three themes of deficiencies emerged from analysis of narratives: Deficiency in implementation of regulatory guidelines; deficiency in disclosure; and deficiency in documentation. Deficiencies lead to patient distrust in surgeons.

### Deficiency in implementation of regulatory guidelines

More than half of the participants were instructed to sign the IC form without any discussion before they underwent surgery, thereby violating guidelines of informing patients of risks, implications, and identity of the surgeon. Participants described big gaps between the time they arrived and the time of admission to surgery. Alona said:The day of the surgery arrived. I was asked to arrive by 11 a.m. I lay there in the corridor by the kitchen as the ward was full and there was no other room. I was shivering, the air-conditioning was on; I was wearing a thin gown … On one hand, I was happy that I am not being operated on, and on the other hand, I wanted already to be 1 minute after surgery. I waited for 4 hours, smelling the odors of disgusting hospital food from the kitchen. I expected to meet the surgeon, but then an attendant came, asked me to sign the form on my chart which was on the bed, and transported me to the operating room.

We thus learn that there was sufficient time to conduct a surgical informed consent discussion, and yet it did not take place. Ron tells us something along the same lines:

They explained nothing to me. Even when I take my dogs to the vet, she explains what she is planning to do and what I should expect thereafter. I was awake; I lay there shivering. There were many people around me, and my chart with the form I needed to sign was on my bed. I signed forms and they came to take me to the operating room.

### Deficiency in sufficient type and amount of disclosure

In their narratives, participants reflected on their expectations of surgeons to update them promptly about the surgery plan, to share information about how they will feel post-operative, the extent of the expected level of pain, and more. Participants also described changes in plan about which they were not informed. Even in cases of high-risk surgeries, where surgeons should provide patients with more detailed information, sufficient disclosure was missing. Ron describes it:

Meanwhile, it was 9 p.m. In the ward they told me: there is an available operating room: You will be admitted next, but if a case of greater emergency arrives, your operation will be moved back. I felt that the expert surgeon was on his way to the operating room. The resident approached me and said, "I will meet you in the operating room soon; I will be there, too". He did not say, “I will be there”, but, "I will be there, too". "They told me the specialist was on his way; I understood that the specialist was on call [Ron takes out a cigarette and lights it, puts the cigarette on the table and keeps silent until the cigarette burns down, as if he is anticipating something]. I was telling myself, "My body is like a container that is going through body work. I was praying that my doctor would have the power to succeed. **…** We waited for the specialist. Another two hours of waiting passed. The resident seemed under stress. I told him that everything would be O.K., to create a positive atmosphere. Little did I know that the specialist had worked from 6 am till the evening, and by then he had already left the hospital. I was seriously out of luck [long period of silence]. It was the first time the resident had performed this type of surgery; he did not know what to do. Everyone understood that he was unsuccessful. I would expect a specialist to be on call 24/7 so that emergency surgeries would not be done by residents with no experience. I need FIVE more surgeries to correct the damage. But the nurses lied to me and said the surgery went O.K.

Some participants were not told that the specialist that they expected to perform their surgery had been replaced by a resident. With regard to this, Anat said:

The surgeon passed by along with a very young doctor and spoke English. "I would like to wish you luck", I said to him. He presented the woman beside him as a resident from Cyprus. "We will cooperate" he said … I was really worried. I was not aware that the hospital trains foreign medical students, and I was thinking, "She does not know the language, she does not know the culture, she is inexperienced … how will she operate on me?!

She was shocked by the change and was distressed, wondering how the resident would be able to perform optimally in a language and culture with which she was not familiar. Some participants were very disappointed that they were not cautioned before the surgery about the high intensity of pain they would experience after it; one of these cases was reported by Alona:

Had I known the chest surgery they performed to remove the tumor in my sternum would be so painful, I would have prepared for it and organized help, borrowed an adjustable chair ahead of time. I could not do anything or even lie down for several weeks, and no one told me about the immense subsequent pain and suffering.

Joseph’s doctor did not inform him about the pain that is caused during a bone marrow aspiration procedure:

The physical therapist and the communication clinician recommended not to do the procedure because my memory and my posture scores were high to begin with. They did not expect the liquid aspiration to improve my movement disorder. A resident surgeon arrived and said, "Get ready; I will do the procedure; it will take 15 minutes". Had we talked about it for a bit, I don't think I would have done it, but a few minutes later he came into my room to do the procedure. The procedure lasted 40 minutes. I was in agonizing pain. He said nothing about how painful it would be.

Gershon shared that he had signed an IC form retroactively without receiving any explanation about the risks of treatment from the surgeon and without him giving authorization to receive the treatment.

My surgeon was great, but her bedside manner was very lacking [Gershon sits erectly upright, and fluently talks about the disease, while crossing his legs]. Two years after the diagnosis, I learned that I was participating in a promising clinical trial with this medication. I received it for two years. I was asked to sign the IC form retroactively, with no discussion and with no consent [nervous hand gestures].

### Deficiency in documentation

Some participants reported no documentation, although the law requires written documentation of the IC discussion. Danit said: “I looked at my chart and I was shocked. It was practically empty with hardly anything documented in it, not even medications and procedures performed”. The empty chart was supposed to include information about the medical situation of Danit, her examination results, medication, and all other treatments she had received. In addition, after an adequate IC discussion, her physician had to write down that an IC dialogue had been conducted. It is self-evident that no documentation of this dialogue had been done as required. The mere lack of documentation of this discussion does not necessarily attest to the absent IC dialogue, though it is most probable that the guidelines of documentation were not executed.

### After discharge: patient trust in surgeons

After discharge, as participants processed and reflected upon their experience, they expressed distrust towards their surgeons. Participants shared their fear of anesthesia, death, and detrimental surgical outcomes. They expected surgeons to address their feelings, but surgeons neither acknowledged their feelings nor alleviated their anxiety and fears. Surgeons’ conduct was short of participants’ expectations, leading to distrust in the surgeon, and extended towards all surgeons. Michelle reflects upon her disappointment:

You are transported stark naked, feeling that you are about to lose it all. You are dust and ashes. In those moments, nothing existed around me and nothing held value. I was all alone, transported yet again to the operating room. No past, no future. Only the huge unease of that moment. I was crying, anxious, fearful, stressed, and disorientated. No one approached me**.** You lie there and feel the nullity of life. Almost everything is erased.

Some participants felt they were transparent; for example, Alona said:

At last my turn arrived. The two surgeons came. I couldn't help but notice that they talked about my procedures like people talk about their daily schedule or flower arrangements. I wondered, it sounds like they know what they are doing, but do they see me? Do they know it's MY cancer? The surgeons didn't introduce themselves. No one talked to me about what was planned for me, what would happen in the operating room, and how I would feel afterwards. No one told me about the agonizing pain I would have post- surgery and after discharge. I am still suffering. I cannot lie down or sit down; I needed an adjustable bed and an adjustable chair, but did not prepare ahead.

Some participants experienced tension regarding their authority to choose a certain option, despite pressure from staff to choose another treatment; Michael provides a description of a typical conflict:

The medical authority is fading and will continue to fade as it crashes against the rock of reality. If every time a procedure is recommended, the surgeon needs my consent on a form, then the medical authority is further diminished and the authority is actually mine. If the surgeon had been confident in the outcomes of the procedure, he wouldn't have needed my signature. Instead, he pressured me to undergo an invasive IV … in order for them to have easier access to my vein. He asked for my permission again and again until I told him very coldly, 'Stop bothering me'.

Gershon participated in a clinical trial without his consent:

They used me as a Guinea-pig for medication for cancer, and I had no idea. I asked the nurse what this medication was, and she said, "The doctor knows what to give you". I looked at what the doctor wrote and asked a family member to check it out on the internet. They checked it out and we found out that it was not a neurological medication. I received the whole debriefing from the net and understood that it was actually chemotherapy. The doctor said nothing about it.

Ron felt that surgeons have no accountability towards patients:

I called the surgeon. He kept walking with an embarrassed expression. He did not approach me [silent]. I am certain that even during the surgery he was on the phone with the senior surgeon who told him what to do. The head nurse stopped the surgeon, and he said, "I know your case. We will need to meet again several times to correct it”. My surgery was a parody. I am waiting in the ward with only a thin gown on my body. My surgeon blocked the blood vessel instead of connecting it. What does he care? In the worst case scenario, there will be gangrene and they will cut my hand off. I will only have one hand … so what?! [Nervous]. Surgeons forgot how to be human beings [low tone]. For all they care, if you lose a hand, nothing happened. You see, it's just a palm, even if he had not saved it, no one would censure him.

Lastly, participants explicitly expressed additional expectations from surgeons; one example is illustrated in Joseph’s words:

I would expect the resident to do a follow-up after the surgery, to come talk to me, to see how I was doing, to check my symptoms and side effects. I did not know if what I felt was expected and normal, or an exception. I was anxious. I felt so dizzy. But he never came. There was another resident surgeon that came to see all his patients. Everyone praised him; he was so humble, too. But my resident, who caused me so much back pain, and thanks to whom I cannot sit or get up, never came by.

## Discussion

The contribution of our study to the understanding of the surgical IC process is threefold: firstly, it is based on narrative interviews, which enabled the solicitation of maximal authentic insights into patients’ experiences; secondly, our study classifies several distinguishable deficiencies in negligent disclosure. Lastly, our insights and understandings provide a basis for proposing practical recommendations as to how to minimize the odds for these deficiencies.

Our disturbing findings, based on participants’ experiences regarding partial IC or lack thereof, provide further evidence of the daily practical difficulty of obtaining surgical IC in reality. We show that patients were deprived of their right to adequate disclosure and involvement. These findings are in line with a previous study [69]. In the chaotic environment of the hospital, the process of surgical IC may seem like nothing but obtaining a patient’s signature [70]. However, it is a pivotal surgeon-patient dialogue that must be planned and integrated into the surgeon’s daily practice [70]. Although the process of IC is indeed challenging in its complex multidimensionality, it sets the stage for patient-surgeon pivotal trust [4]. Participants attested that surgeons infringed upon their rights of surgical IC, although they had the mental capacity and were communicative and environmentally aware. Learning that patients’ consent was not adequately solicited for our participants, we argue that these surgeons had nullified the meaning of informed consent.

Our findings attest to the infringement of patients’ right to disclosure. Most patients signed the IC form without receiving any information. Surgeons neglected the obligation of disclosure: They did not receive true IC, as they did not conduct a discussion with patients explaining the required elements; they did not check patients’ understanding of the procedure; did not answer their questions; did not examine patients’ understanding of potential ramifications of the procedure. They did not discuss alternatives, rates of success and limits of current medical knowledge regarding possible side effects [2, 71]. This observation corroborates previous scholarly understandings that, “Rules and standards of care are easily recognized, and are widely disseminated, but professionals consistently and even brazenly disregard them” [20]. There should be no tolerance among hospital directors for nullifying patients’ right to the full and appropriate process of surgical IC.

Inspired by Kantian theory, we argue that surgeons may be involved in wrong pro tanto conduct and may not be fully committed to the principles of IC. They functioned in a paternalist manner as if they were surrogates for participants who, in fact, needed no surrogate. These paternalists may have concentrated on medical facts and made decisions based on their own knowledge, values, and judgments about what treatment should be done, thus violating patient autonomy [17]. The respect for autonomy and its subsequent disclosure guidelines are based on the premise that patients’ personhood must be recognized, and that asymmetrical patient-clinician power relations should be transformed into egalitarian power-sharing [72]. “Pastoral power” is a term coined by Foucault [73] to describe an aspect of productive power which is exercised during the provision of care to another person. He attached the metaphor of shepherd (rather than king) to this form of power, which entails the protection and direction of the ‘flock,’

Foucault distinguished between four dimensions of pastoral power, of which two are highly relevant for the analytical analysis of surgical IC process. The first is the ‘aim of salvation’ which pre-assumes that redemption from suffering is of upmost importance; the second, ‘power which commands and sacrifices’ relates to the authoritative stance of clinicians when they ‘command’ treatments or examinations. Since IC discussion involves an overt verbal dimension as well as a covert aspect of the patient-clinician power relationship, various informed choices, as well as results of the IC experience, can stem from different IC dialogue narrations and the level of power symmetry between the parties.

In contrast to a long-term patient-primary physician relationship, where if dissatisfied the patient may transfer to a different physician, the encounter of the patient with the surgeon occurs only on the day of the surgery. Prior to and during this day, the patient is likely to be anxious, vulnerable and suffering; therefore, the patient-surgeon relationship is much more asymmetrical, and the surgeon may have much more power than does the patient. The power stance of the surgeon as predisposed for the salvation of the patient, together with the authoritative power to ‘command’ the surgery, converge toward a more autocratic surgery while downplaying the significance of the IC dialogue and expropriating the little power the patient [still] possesses. Unfortunately, the result of this asymmetric relationship of power may constitute a paternalistic attitude toward the patient, as reflected in a flawed IC process, which is a result of what Corrigan [74] has dubbed ‘empty ethics’.

More than half our participants were instructed to sign the IC form after a long waiting time and just a few minutes before going into the OR. Participants expected disclosure regarding the post-op recovery, discomfort, and pain. Treating patients as if they were non-human objects reflects an attitude of objectification; **objectification** is the act of treating a person, or sometimes an animal, as an object or thing. It involves dehumanization, i.e., disavowing the humanity of another person. According to philosopher Martha Nussbaum [75], a person is objectified by other(s) if one of seven criteria occurs; analysis of the narratives indicates that the following dimensions of objectification were in place in the lived experience of our informants: Denial of autonomy – treating the person as lacking in autonomy or self-determination; Inertness – treating the person as lacking in agency or activity; Denial of subjectivity – treating the person as though there is no need for concern for their experiences or feelings. Langton [76] added three dimensions to Nussbaum’s classification; one of them is ‘silencing’ - which is highly relevant to our analysis, as it is evident in our informants’ narratives; Silencing denotes a treatment of a person as if they were silent, lacking the capacity to speak.

This view of objectification of the participants during the IC process is congruent to that of Corrigan’s [74], who claimed that “patients often become objects of the bureaucratic machinery.” (p. 786). Hence, some surgeons fail to fulfill the positive obligation required by the principle of respect for autonomy, thus resulting in the objectification of patients and their patronization [20]. We contend that the nullification of IC, as a manifestation of ‘empty ethics’ in Corrigan’s [74] words, may be perpetuated and preserved by the following inherent paradoxes underlying this conduct.

On the one hand, there is the supreme professional core value that constitutes the obligation of surgeons to use their knowledge and skills to save lives. Indeed patients in acute care may fail to give sufficient weight to relevant medical facts and make incorrect judgments about what is best for them. Thus, despite the need to respect patient autonomy, it may not always be best to make a shared doctor-patient decision [17].

On the other hand, there are the ethical values behind the golden standard of respect for autonomy which obligate surgeons to carry out a surgeon-patient dialogue and allow the patients to freely decide and choose among treatments to be performed on them. These two co-existent values, the professional value and the ethical value, may contradict each other, and may create a duality resulting in partial, insufficient, or total lack of disclosure regarding patients’ participation in decision making.

It is also possible that physicians experience a dilemma regarding the depth of disclosure: On one hand, they may want to provide all available information so they are not vulnerable to litigation, and on the other hand, they may reduce disclosure to alleviate patient anxiety [4]. Negligent disclosure may seem to be a solution to this dilemma. This solution is embedded in the context of asymmetrical power relations between the surgeon and the patient, which entail powerful autocratic medical practices such as negligence [74]. Participants felt that they became objects of the bureaucratic machinery of surgery.

The deficiencies in the process of surgical IC did not meet participants’ expectations, and this disappointment led to distrust. Participants expected surgeons to act exclusively in their best interest, not only while operating on them, but also outside the operating room. They expected surgeons to discuss alternatives and to inform them about what to expect after their surgery and discharge. But most participants were unclear about how they would function after surgery, the extent of pain they could expect, and the discomfort they might experience after discharge.

In addition, participants reported fear of anesthesia and death. Anxiety and fear in patients increase as the severity of their surgery increases [56]. The participants’ fear and the failure of their surgeons to alleviate this anxiety may have inhibited effective recuperation [77, 78]. Participants’ expectations for communication and accountability were not met and created distrust in surgeons. This wrong conduct by surgeons breached participants’ trust in all surgeons and created high levels of tension, anxiety and distress, phenomena that are acknowledged to carry greater morbidity risks in 30 to 40% of the cases after surgery [79].

This retrospective realization made participants feel deceived. The similar pattern of wrongful conduct in these upsetting experiences across hospitals and surgeries leads us to contend that the existing practice of unintentionally nullifying IC does not stem solely from lack of communication skills or patronization. Similar to Harrison and Taylor [80], we contend that findings may reflect organizational values in the hospital that the management supports in order to facilitate shorter waiting times and increase the number of surgeries. Managements and surgeons may view the IC process as consuming valuable time and irrelevant for patient decision-making in life-saving surgeries [3]. Our findings, however, indicate that the IC process is important for patients’ well-being. While the limitations of the IC process may evoke cynicism among surgeons, participants explicitly expressed their need of disclosure to relieve their anxiety; to receive answers to their questions; and to shape realistic expectations regarding their post-operative condition. Had the disclosure taken place and included these pieces of information, patient-surgeon distrust would have been avoided.

**Fig. 1:**
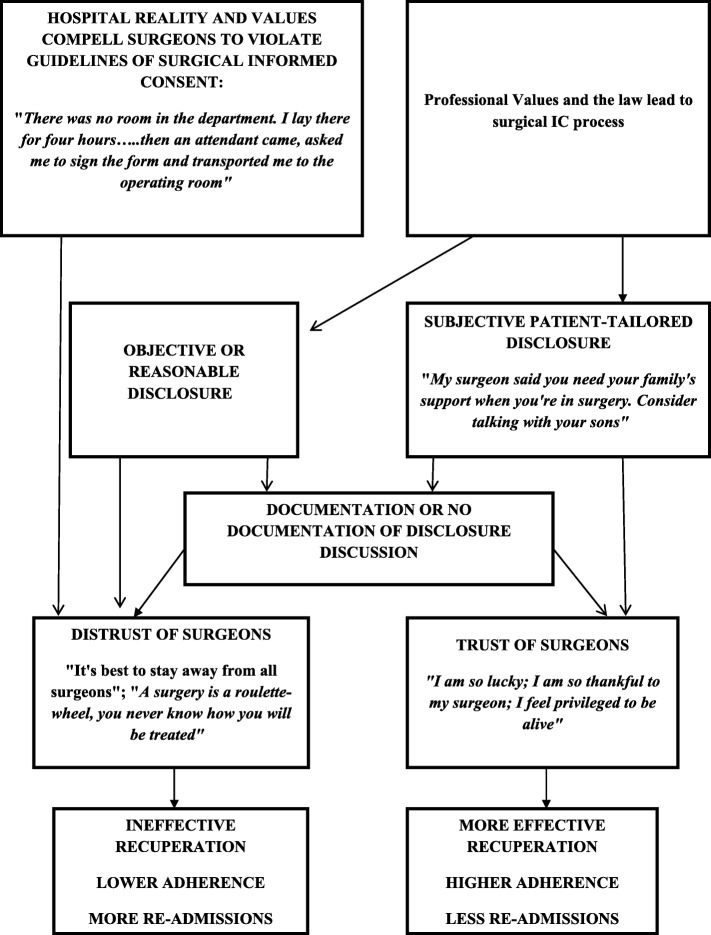
A Trust Building Multi-Phase Model of Surgical Informed-Consent Process

The combination of surgeons’ knowledge, training, expertise and professional values with inputs of the patient will allow a discussion that reduces paternalism and objectification as surgeons will avoid making value judgments that vary from person to person about what is best for their patients and present rational arguments regarding what they are advocating as the best course [17, 47]. Such disclosure in the IC process may allow the surgeon to offer interventions that are beneficial and desired by the patient [81], thereby enhancing the surgeon-patient interactions and create trust [8]. Figure 1 presents a model of surgical IC process which facilitates surgeon-patient trust, based on the insights we gained after analyzing the narratives.

### Research limitations and future studies

Since our informants were anxious and tense during the waiting time before the surgery, they might have not fully remembered all communication with the staff, and therefore underestimated the amount of information provided during the conversation with the surgeon. Also, the relatively small number of participants may not have reflected a sufficient variety of experiences [82]. Future studies should focus on ways to improve the appropriate implementation of the IC law, as well as requesting surgeons to reflect upon their own experience of IC conversations.

### Practical recommendations

Compared to patients, surgeons have always had greater knowledge of the effects of medical treatment and this may have fostered a belief that they should decide which treatments are appropriate for patients [81]. In order to meet the prima facie right of respect for autonomy, moral agency, and human dignity in a constrained work environment at hospitals, we offer the following recommendations to advance the ethical and responsible implementation of IC rules. Changes must take place at both the organizational and team levels. Firstly, organizational and professional values regarding surgical IC may be mapped and clarified as they were mapped for community health organizations [80]. Second, organizational quality measures should ideally include the assessment of proper IC disclosure as experienced and reported by patients. This would enhance the appropriate incorporation of ethical standards in projects of quality improvement, a neglected area in quality improvement initiatives [83]. The process of IC is to be seen as an on-going process rather than a discrete act of choice that takes place in a given moment of time [84].

Third, organizational development is required for eradicating the prevalent culture of objectification at hospitals. Fourth, hospital managers should require medical staff to consistently renew their commitment to respect patient autonomy and properly implement IC guidelines through interventions at the team level. At the team level, students, residents, and specialists should participate in training aimed at internalizing the ethical values underlying IC and acquiring new skills for patient-tailored surgeon-patient discussions regarding IC.

Patient-tailored disclosure can provide the surgeon with a structure of “talking points” that encourage personalized, comprehensive discussions that are appropriate for the patient’s concerns” [8]. Because of the great importance given to personal interaction and the addition of the personal touch for building trust relations between patients and staff, dialogue skills regarding values and preferences of the patient are crucial [85]. Workshops on IC patient-tailored discussion should be part of the clinical curriculum at schools of medicine and of continuing education at hospitals. Patient-tailored disclosure dialogues should also aspire to be socio-culturally tailored to the background of each patient [86]. Moreover, acknowledgement of patient’s fears may allow surgeons to better alleviate the anxiety by adjusting the IC process for each patient. Team members are called upon to identify groups of patients according to surgical risks and to follow patient-tailored surgical IC disclosure [69].

## Conclusions

Having edged back from either soft or hard paternalism, it is now time for a renewed effort to construct a conscious, practical, trust-facilitating process of surgical IC [87]. It is our hope that the model we propose may offer insights which could serve as the cornerstone for such an improved surgical IC process.

## Data Availability

Authors have no permission to share the raw data.

## Abbreviations

COLLMAN: College of Management Academic Studies
IC: Informed consent
